# Mechanism of Sleep Disturbance in Children with Atopic Dermatitis and the Role of the Circadian Rhythm and Melatonin

**DOI:** 10.3390/ijms17040462

**Published:** 2016-03-29

**Authors:** Yung-Sen Chang, Bor-Luen Chiang

**Affiliations:** 1 Department of Pediatrics, Taipei City Hospital Renai Branch, Taipei 106, Taiwan; yschang1018@gmail.com; 2 Graduate Institute of Clinical Medicine, College of Medicine, National Taiwan University, Taipei 100, Taiwan; 3 School of Medicine, National Yang-Ming University, Taipei 112, Taiwan; 4 Department of Medical Research, National Taiwan University Hospital, Taipei 100, Taiwan

**Keywords:** melatonin, circadian rhythm, sleep disturbance, atopic dermatitis (AD)

## Abstract

Sleep disturbance is common in children with atopic dermatitis (AD). It is a major factor leading to impaired quality of life in these patients and could have negative effects on neurocognitive function and behavior. However, the pathophysiology of sleep disturbance in children with AD is poorly understood, and there is no consensus on how to manage sleep problems in these patients. Pruritus and scratching could lead to sleep disruption but is unlikely the sole etiology. The circadian rhythm of cytokines, the immune system, and skin physiology such as transcutaneous water loss and skin blood flow might also play a role. Recent studies have suggested that melatonin could also be involved due to its multiple effects on sleep, immunomodulation, and anti-oxidant ability. Environmental factors should also be considered. In this review, we summarize the current understanding of the pathophysiology of sleep disturbance in children with AD, and discuss possible therapeutic implications.

## 1. Introduction

Atopic dermatitis (AD), also known as eczema, is a common chronically relapsing pruritic inflammatory skin disease affecting 15%–30% of children, and its prevalence is continuously increasing [1]. AD is a complex disease with a genetic predisposition strongly influenced by innate and adaptive immune responses, as well as environmental factors, and to this day the pathophysiology is still not fully understood [2]. Disturbed sleep is reported in 47%–60% of patients and is a major factor leading to an impaired quality of life [3,4,5]. The majority of previous studies evaluated sleep as a secondary focus in questionnaires assessing the severity or quality of life in patients with AD, and most relied on a single item typically describing global sleep quality [3]. Few studies have used laboratory-based polysomnography (PSG), the gold standard in sleep examinations, to assess sleep in this group of patients [6,7,8], probably due to the inconvenience of having to be performed overnight at a sleep center and having to attach multiple leads and equipment which may cause more skin irritation. Actigraphy is a small, wrist-worn device that estimates sleep-wake patterns by using activity-based monitoring. It has been validated to delineate sleep parameters highly correlated with laboratory-based PSG in children with AD [9] and is increasingly used to provide objective sleep assessments in these patients due to its ease of use. Objective measurements showed that children with AD have significantly reduced sleep efficiency, longer sleep onset latency, more sleep fragmentation, and less non-rapid eye movement sleep [9]. Sleep disturbance can have many negative consequences, including impaired neurocognitive function, higher rates of behavioral problems, and changes in mood [10,11]. It has been suggested that AD is associated with attention deficit hyperactivity disorder, emotional and conduct problems, and short stature only when accompanied with sleep problems [12,13,14]. With growing recognition of the importance of addressing sleep disturbance in children with AD, the practice guidelines from the American Academy of Allergy, Asthma and Immunology (AAAAI), the American College of Allergy, Asthma and Immunology (ACAAI), and the American Academy of Dermatology (AAD) have all recommended routinely assessing sleep in these patients [15,16]. However, the pathophysiology of sleep disturbance in children with AD is poorly understood, and there is no consensus on how to manage sleep problems in these patients. Recent studies have suggested that melatonin may have a role in the sleep disturbance in children with AD [9,17], and could possibly be used for treatment [18,19]. We performed a comprehensive literature search in PubMed/MEDLINE for all articles with the key words “atopic dermatitis” or “eczema” and “sleep” published before 1 February 2016. We also reviewed relevant articles retrieved from references. In this review, we describe our current understanding of the mechanism of sleep disturbance in children with AD and the possible role of circadian rhythm and melatonin, and discuss treatment implications.

## 2. Pruritus and Scratching

Perhaps the most straightforward reason for sleep disturbance in children with AD is thought to be pruritus and scratching movements disrupting sleep. Disease severity is strongly associated with sleep disturbance in children with AD. The Scoring Atopic Dermatitis Index (SCORAD) is a commonly used measure of the severity of AD with scores ranging from 0 to 103 and greater scores indicating worse symptoms. It was found that a SCORAD of higher than 48.7 predicted poor sleep efficiency with high sensitivity (83.3%) and specificity (75%) (area under the curve = 0.81, *p* = 0.001) [9]. It has been suggested that the cause of itch in AD is neuropeptide-mediated vasodilation and change in skin temperature [3], and sensory hypersensitivity [20], as neuroselective transcutaneous electrical stimulation preferentially evokes itch in patients with AD in contrast to healthy subjects [21]. The cause of sensory hypersensitivity might be due to eosinophils inducing cutaneous nerve growth [22], or increased skin levels of nerve growth factor [23]. The strongly pruritogenic interleukin (IL)-31 is a cytokine produced by T cells that increases the survival of hematopoietic cells and stimulates the production of inflammatory cytokines by epithelial cells [1]. IL-31 has been suggested to be a major factor inducing pruritus in AD [1], since both IL-31 and its receptor are overexpressed in lesional skin [24,25]. Brain-derived neurotrophic factor and substance P have also been suggested to have a role in the pruritus in AD [26]. The itch in AD is often worse at night, which could lead to sleep disturbance. The severity of pruritus is difficult to observe and is usually described by the patient’s subjective scoring. This makes evaluation of pruritus in sleep challenging, especially in children. Some studies showed that pruritus severity scoring correlated with sleep problems in children with AD [9,27], but some showed poor association [28]. Another study showed that sensory hypersensitivity was correlated with lower sleep quality in children with AD [29]. Serum IL-31 level was found to be associated with subjective sleep loss and decreased stage N1 sleep [9,30], but did not correlate significantly with pruritus severity score [30].

Compared with itch, more objective tools can be used to evaluate scratching, such as polysomnography or actigraphy with/without infrared video monitoring. Studies using infrared video monitoring to identify scratching events found that the percentage of time in sleep with scratching movements ranged from 4.7% to 14.3% in patients with AD [31,32]. Scratching occurred mainly in stages N1 and N2 sleep [33], but there was also significantly more limb movements during the deep sleep stage N3 in children with AD compared with controls [9]. Studies have reported that scratching and movements in sleep are correlated with higher disease severity, lower sleep efficiency [9,34], and more sleep disruption [35], However, Reuveni *et al.* [6] reported that scratching accounted for only 15% of arousals and awakenings in children with AD, and our group also found that arousals caused by limb movement were not significantly correlated with sleep efficiency in the patients [9]. Therefore, the current evidence supports that itch and scratching movements play a part in the sleep disturbance of children with AD, but are unlikely the sole cause.

## 3. Cytokines and Immune Cells

Sleep and the circadian rhythm have complex relationships with immune function and cytokine production. These systems are probably temporally integrated to respond to environmental changes and optimize adaptation [36]. Diurnal patterns have been shown to exist in immune cell counts, immune cell function, and cytokine levels. Total leukocyte numbers, memory T cell number, and naive T cell count and proliferative function peak at night, while NK cell number and activity has a rhythm with a daytime maximum [37,38]. Naturally occurring regulatory T cells are also at the highest levels at night and express maximum suppressive activity at 2 a.m. and almost no suppressive activity at 7 a.m. [39]. Pro-inflammatory cytokines such as IL-1β, IL-2, tumor necrosis factor-α, interferon-γ, and IL-6, are elevated at night and generally promote sleep, while anti-inflammatory cytokines such as IL-4 and IL-10 are induced after awakening and could inhibit sleep [38,40]. Immune response to lipopolysaccharide stimulation [41], vaccination [42], and susceptibility to infection [43] also vary according to the timing of stimulation and can be altered with sleep loss [36]. Sleep deprivation could result in a shift in the Th1/Th2 balance toward Th2 dominance [44] and could disturb the functional rhythm of regulatory T cells [39].

Many cytokines and immune cells are involved in the pathogenesis of AD, and it is possible that they overlap or interact with those which regulate or influence sleep. The mechanism of AD is complex, with an initial Th2 phase and a chronic Th1-predominant phase. Recent studies showed that Th17 and Th22 cells might also play a role in the acute and chronic phase of AD, respectively [45,46,47]. Elevated pro-inflammatory cytokines and T cell activity at night might contribute to pruritus and flares inducing sleep disturbance in AD, but direct evidence is lacking and much is still unclear. Only a few studies have investigated the role of cytokines and chemokines in the sleep disturbance in AD patients. Bender *et al.* [33] reported a study of 20 adults with AD and found that the morning-evening change of IL-6 production by peripheral blood mononuclear cells stimulated with anti-CD3 was correlated with sleep efficiency measured by actigraphy, with improved sleep in those with increased differential between morning and evening IL-6 production. A study of 24 Chinese children by Hon *et al.* [28] showed that wrist activity during sleep was correlated with plasma concentrations of cutaneous T-cell attracting cytokine (CTACK), macrophage-derived chemokine (MDC), and thymus and activation regulated chemokine (TARC), but was not correlated with subjective pruritus or sleep loss. Our group studied 72 children with AD aged 1 to 18 years and found that a higher morning serum IL-4 level was weakly correlated with a higher sleep efficiency measured by actigraphy (*r* = 0.28, *p* = 0.02). Morning serum levels of IL-6, IL-1β, and IL-10 were not correlated with sleep parameters. The ratio of interferon-γ and IL-4 (interferon-γ:IL-4) was significantly lower in the subgroup of patients with poor sleep efficiency (*p* = 0.01) [9], compatible with the theory that sleep loss could cause a shift in the Th1/Th2 balance toward Th2 dominance [44]. Summarizing the current evidence, the relationship between atopic dermatitis, cytokines, and sleep disturbance cannot be clearly described. This might partly be due to the diurnal rhythm of cytokine levels which make interpretation of single measurements in clinical studies challenging. Further studies are needed to delineate these intertwined relationships.

## 4. Circadian Rhythm

In addition to the effects on cytokines and immune cells mentioned above, the circadian rhythm might also influence AD in several other ways. Cortisol levels are the highest in the morning, gradually declines, and reaches trough levels in the evening after sleep onset. It was suggested that this diurnal pattern contributes to increased pruritus at night in AD and other pruritic skin diseases because the anti-inflammatory effect of cortisol is at its minimum during this time [48,49]. A deviation of the circadian cortisol rhythm in AD patients was found in a study by Heubeck *et al.* [50]. However, studies directly investigating the relationship between cortisol levels and sleep disturbance in children with AD are lacking.

Skin cells express circadian clock genes, such as CLOCK (circadian locomotor output cycles kaput), BMAL1 (brain and muscle aryl hydrocarbon receptor nuclear translocator (ARNT)-like protein-1), and Period1-3 (Per1-3), which have autoregulatory feedback loops and maintain a rhythm of oscillating gene products [51]. Studies have reported various skin physiology showing circadian rhythmicity, such as transepidermal water loss (TEWL), skin permeability, skin surface pH, and skin temperature [52]. Higher TEWL in the evening has been associated with higher itch intensity in AD [48]. Skin blood flow rates also have circadian rhythmicity. The blood flow rate is lowest in the morning and highest in the afternoon and early evening, and has a second peak in the late evening before sleep onset [53]. Yosipovitch *et al.* [53] found that this rhythm is maintained in irritated skin under topical steroid treatment. However, topical steroid significantly decreased the blood flow of irritated skin at night but not in the daytime. Viewing the decrease of blood flow in irritated skin as the therapeutic effect of topical steroids, they suggested that their results could imply that dosing topical steroids at night might be advantageous.

## 5. Melatonin

Melatonin is a hormone secreted by the pineal gland that is essential for regulating sleep and the circadian rhythm. It is secreted with a diurnal pattern: Secretion increases soon after the onset of darkness, peaks in the middle of the night (between 2 and 4 a.m.), and gradually falls during the second half of the night [54]. Extrapineal melatonin has also been detected in multiple tissues such as the skin, lymphocytes, mast cells, airway epithelium, brain, retina, gastrointestinal tract, and reproductive tract [55]. Various types of skin cells and lymphocytes produce melatonin and express melatonin receptors [55,56]. Recent studies have suggested that melatonin plays a role in AD due to its multiple effects on sleep, immunomodulation, and anti-oxidant ability [17,18]. 

Melatonin has a sedative effect, which could be due to a direct phase-shifting effect on the suprachiasmatic nucleus, the master clock controlling circadian rhythms, or its effect of decreasing the core body temperature [57,58]. Schwarz *et al.* [59] studied the rhythmic behavior of melatonin secretion in patients with AD by measuring melatonin serum levels every 2 h, and found that the circadian melatonin rhythm was abolished or diminished in most patients. Only 4 of the 18 patients showed a normal secretion pattern of melatonin. In a study of 72 children with AD and 32 healthy controls, our group found that nocturnal melatonin secretion, measured by morning urinary 6-sulfatoxymelatonin levels, was higher in AD patients compared with controls. We also found that in patients with AD, a higher nocturnal melatonin level was associated with better sleep efficiency, less sleep fragmentation, and milder disease severity [9]. Sleep deprivation could result in increased melatonin secretion [60,61]. Therefore, a potential explanation of our findings is that AD patients with sleep disturbance could have a compensatory increase in nocturnal melatonin secretion, and those who respond to this compensatory increase benefit from its effect in improving sleep and skin condition. It is also possible that melatonin plays a role in the pathogenesis of AD.

Melatonin also has immunomodulatory, anti-inflammatory, and anti-oxidative effects [17,62,63], which might improve the skin inflammation and help maintain a functional epidermal barrier in AD patients [17]. Various immunomodulatory mechanisms of melatonin have been reported. Th1 and Th2 cells express membrane and nuclear receptors for melatonin; via these receptors, melatonin induces the synthesis of IFN-γ, IL-2, IL-6, and IL-12 by lymphocytic and monocytic cell lines and amplifies melatonin receptors [17,64,65]. Melatonin could also influence the activity of NK cells, T and B lymphocytes, granulocytes, monocytes, and mast cells [17,66]. Some studies found that melatonin had an immunostimulatory function, while others reported its anti-inflammatory effects. Therefore, it was suggested that melatonin acts as an immune buffer, acting as a stimulant under basal or immunosuppressive conditions, and exhibiting anti-inflammatory function in states of acute inflammation [67]. However, whether and how melatonin modulates the complex inflammatory pathways in atopic dermatitis has not been extensively explored.

Melatonin efficiently neutralizes several free radicals and stabilizes cell membranes through the upregulation of antioxidant enzymes [68]. It has been shown to be protective against the ultraviolet radiation-induced skin damage including DNA repair [69], and has been reported to help ulcer healing in patients with gastroesophageal reflux disease and peptic ulcer disease [70]. Use of melatonin has also been implicated in improving androgenetic alopecia [71,72], and could possibly promote wound healing [73]. Whether the antioxidant abilities of melatonin play a role in AD is still unclear.

Currently, only a few studies have investigated the effect of exogenous melatonin in AD patients. An animal study showed that melatonin suppresses the development of AD-like dermatitis in DNFB-treated NC/Nga mice by reducing total serum immunoglobulin E level and Interleukin (IL)-4 and Interferon-γ production by activated CD4^+^ T cells [74]. Our group conducted a randomized, double blind, placebo-controlled trial to investigate whether melatonin is effective for improving skin inflammation and sleep in children with atopic dermatitis aged 1 to 18 years. We found that 3 mg of oral melatonin before bedtime for four weeks significantly improved AD disease severity, on average lowering the disease severity score, the SCORAD index by 9.1 compared with after a placebo (95% CI, −13.7 to −4.6; *p* < 0.001), from a mean (SD) of 49.1 (24.3) to 40.2 (20.9). Melatonin also significantly shortened the sleep onset latency by 21.4 min after melatonin treatment compared with after a placebo (95% CI, −38.6 to −4.2; *p* = 0.02). The improvement in the SCORAD index did not correlate significantly with the change in sleep-onset latency (*r* = −0.04; *p* = 0.85) [18]. This supports that, in addition to sleep-promoting effects, other properties of melatonin, such as immunomodulation or anti-oxidation, could play a role in modulating the sleep disturbance in AD patients.

## 6. Environmental Factors

The total serum IgE level has been reported to be associated with pruritus and sleep loss in patients with AD [9,75,76]. Our group found that serum allergen-specific IgE levels to dust mite Derp and Derf were significantly correlated with sleep disturbance in children with AD, including decreased sleep efficiency, higher percentage of time awake during sleep, and more sleep fragmentation [9]. Dust mites are one of the most common allergens that induce allergic sensitization in children [77]. Pillows and mattresses are major reservoirs of dust mite allergens, so the sleeping environment could serve as a main source of dust mite exposure. Our study results support that allergic sensitization and exposure to dust mites during sleep could also contribute to sleep disturbances in AD patients. Cosleeping with the parents is common in children with AD due to their skin condition and has been reported to be associated with more severe disease and sleep disturbance of parents [78]. In healthy children, cosleeping has also been found to be a predictor of night wakings [79]. However, these were association studies, and it is unknown whether cosleeping behavior may also contribute to the sleep disturbance in AD.

## 7. Implications for the Treatment of Sleep Disturbance in AD

Sleep is usually viewed as a secondary focus of disease control in studies assessing therapeutic effects on AD, and few studies have evaluated treatment methods specifically aimed at improving sleep in patients with AD. There is currently no consensus on the management of sleep disturbance in children with AD, and most treatment strategies are based on expert opinion [19,80]. First-generation antihistamines are most commonly used for sleep problems in AD patients, most likely due to their sedating effect [19], as they can cross the blood-brain barrier and affect histamine’s role in maintaining central nervous system arousal [80]. However, tolerance often occurs after four to seven days of treatment, and the sedating effect vanishes, limiting its usefulness [81,82]. Anticholinergic side effects such as blurred vision and dry mouth are also major concerns. Other suggested pharmacologic treatment aimed at promoting sleep in AD patients including benzodiazepines, chloral hydrate, and clonidine all lack supporting evidence [19,80]. Benzodiazepines have sedating and anxiolytic effects, but they carry the risks of tolerance to sedating effects, rebound worsening of sleep problems on discontinuation, and addiction. Negative side effects including muscle relaxation and memory problems also make them less favorable for use in children [80]. The fact that benzodiazepines could suppress nocturnal melatonin secretion [83] might also be a detrimental aspect of their use if melatonin truly has an important role in AD. Chloral hydrate use has been discontinued in the United States due to risks of hepatotoxicity and respiratory depression [19].

**Figure 1:**
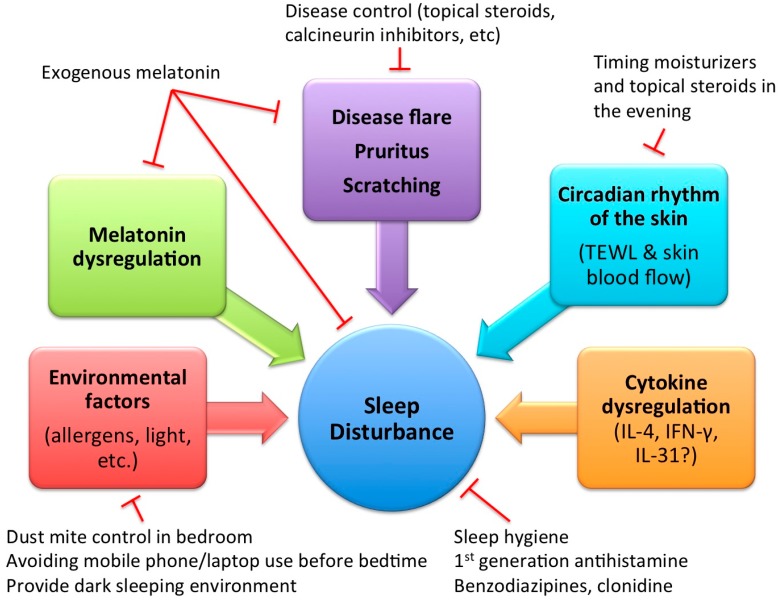
Mechanism of sleep disturbance in atopic dermatitis and possible treatment implications. T arrows indicate possible inhibitory effect with the suggested management options. TEWL: transepidermal water loss; IL-4: interleukin-4; IFN-γ: interferon-γ.

From our review of the mechanism of sleep disturbance in children with AD, several treatment strategies could be implicated (Figure 1). Pruritus and scratching play an important role and therefore disease control, such as with topical steroids or calcineurin inhibitors, is crucial in improving sleep disturbance in AD, but might not be sufficient [19]. Antihistamines have not been demonstrated to improve pruritus in AD in randomized controlled trials [84], and evidence for use of other anti-pruritic medication is currently limited. IL-31 is a potential target, but further studies are needed. Melatonin has sleep-promoting, immunomodulatory, and antioxidant properties, and it is a promising treatment strategy, as it has been demonstrated to improve both sleep onset and disease severity in children with AD in a randomized controlled trial [18]. Melatonin also has a good safety profile with minimal adverse effects and does not have addictive or withdrawal concerns, making it a favorable choice in children [85]. Further studies are needed to evaluate the optimal dose and duration of melatonin treatment.

Regarding the effects of circadian rhythm and environmental factors, applying moisturizers and topical steroids in the evening might be advantageous, as the TEWL is highest at this time, and skin blood flow rate is most affected by topical steroids [49]. Dust mite sensitization might have a role in the sleep disturbance in AD patients [9]; therefore, regular cleaning and measures to avoid dust mite accumulation in the sleeping environment should be encouraged. Light, especially blue light, suppresses melatonin secretion. Therefore, it should be important to provide a dark sleeping environment devoid of blue light from mobile phones and computer screens. Other general sleep hygiene measures, such as developing positive and consistent bedtime routines, should also be recommended, but sleep-directed behavioral therapies have not been evaluated in improving sleep problems in AD patients [49].

In summary, the pathophysiology of sleep disturbance in patients with AD is complex and likely involves intertwined relationships between sleep, the circadian rhythm, the immune system, and the environment. Further studies are needed for a better understanding of these relationships and for building better treatment strategies accordingly.

## Abbreviations

AD	atopic dermatitis
IL	interleukin
PSG	polysomnography
SCORAD	Scoring Atopic Dermatitis Index
TEWL	transepidermal water loss
